# Protein Kinase C Isozymes and Autophagy during Neurodegenerative Disease Progression

**DOI:** 10.3390/cells9030553

**Published:** 2020-02-27

**Authors:** Humeyra Nur Kaleli, Ebru Ozer, Veysel Ogulcan Kaya, Ozlem Kutlu

**Affiliations:** 1 Molecular Biology, Genetics and Bioengineering Program, Sabanci University, 34956 Istanbul, Turkey; hkaleli@sabanciuniv.edu (H.N.K.); ebruozer@sabanciuniv.edu (E.O.); vogulcan@sabanciuniv.edu (V.O.K.); 2 Nanotechnology Research and Application Center (SUNUM), Sabanci University, 34956 Istanbul, Turkey; 3 Center of Excellence for Functional Surfaces and Interfaces for Nano Diagnostics (EFSUN), Sabanci University, 34956 Istanbul, Turkey

**Keywords:** Protein kinase C, autophagy, neurodegeneration, polyphenols

## Abstract

Protein kinase C (PKC) isozymes are members of the Serine/Threonine kinase family regulating cellular events following activation of membrane bound phospholipids. The breakdown of the downstream signaling pathways of PKC relates to several disease pathogeneses particularly neurodegeneration. PKC isozymes play a critical role in cell death and survival mechanisms, as well as autophagy. Numerous studies have reported that neurodegenerative disease formation is caused by failure of the autophagy mechanism. This review outlines PKC signaling in autophagy and neurodegenerative disease development and introduces some polyphenols as effectors of PKC isozymes for disease therapy.

## 1. Introduction

Extracellular signaling molecules bind to their specific receptors and alter the intracellular amount and distribution of the secondary messenger molecules within the cytoplasm. These secondary reporter molecules act on target proteins, which consequently control the gene expression. The most common reversible protein phosphorylations involved in the control of gene expression are Serine (Se), Threonine (Thr) and Tyrosine (Tyr) phosphorylation.

Protein Kinase C (PKC) protein family is a phospholipid-dependent serine/threonine kinase discovered by Nishizuka and his colleagues in the 1970s. This protein family was initially defined as PKM due to Mg^2+^ dependent activation, but later renamed, PKC due to Ca^2+^ dependent activation [1]. The protein kinase family consists of over 15 subgroups with more than 500 kinases, each of which is involved in the regulation of gene expression; thereby, the downregulation or upregulation of these kinases induces severe consequences in the progression of disorders including neurodegenerative diseases [2,3,4,5,6,7].

Autophagy is a highly conserved cellular degradation machinery, essential for survival, differentiation, development, and cellular homeostasis. This mechanism functions under basal conditions and becomes activated under conditions of cellular stress, such as nutrient limitation, oxidative stress or abnormal protein accumulation [8]. Autophagic pathway is initiated by the formation of double or multi-membrane vesicles in the cytoplasm. These vesicles engulf portions of the cytoplasm containing the cargo and carry them to the lysosome. After the fusion of the autophagic vesicles with lysosomes, the cargo is degraded, and its constituents are recycled [9]. Autophagy-related genes (ATG) genetically regulate this pathway and to date, more than 30 ATG genes have been reported. The encoded proteins of these genes interact with different signaling pathways and serve a protective role for organisms against several pathological conditions including neurodegeneration [10,11,12,13].

Neurodegeneration is the progressive loss of structure or function of neurons and usually results in neuronal cell death, which is in fact the main cause of debilitating, incurable neurodegenerative diseases. The aggregation of abnormal proteins is thought to be a primary reason for the development of many neurodegenerative diseases. Therefore, autophagic activity is thought to affect disease progression [14,15]. Moreover, the association between PKC with neuropathological conditions has been are described in several studies [16,17,18,19,20,21,22,23,24,25,26,27,28,29]. However, the importance of autophagic pathways and its interaction with PKCs in the development of neurodegenerative diseases is still being debated. In this review, first, we summarize the molecular mechanisms and the physiological relevance of PKC and autophagy. Then, we review how autophagy and PKCs are involved in the pathology of certain neurodegenerative diseases.

## 2. PKC Superfamily

PKC is a subgroup of the kinase family and comprises ten members. The distinguishing feature of PKCs is that they include an N-terminal regulatory domain connected to a C- terminal catalytic domain through a hinge domain [30,31]. Each of the PKC isozymes share common structural characteristics since they have four conserved domains, C1, C2, C3 and C4, C1 and C2 are located on the N-terminal regulatory domain while C3 and C4 reside on the C-terminal catalytic domain. The C1 domain structure shows that it has a hydrophilic ligand binding site enclosed with hydrophobic amino acids. On the hydrophilic region, there are diacylglycerol (DAG) and phorbol esters binding sites [32]. C2 includes a secondary messenger, Ca^2+^, binding site [33]. C3 has an ATP binding site and C4 has protein substrate binding sites [34]. All PKC isozyme have a pseudosubstrate region that is a substrate-mimicking short amino acid sequence which binds the substrate-binding cavity in the catalytic domain, rendering the enzyme inactive [35].

**Figure 1:**
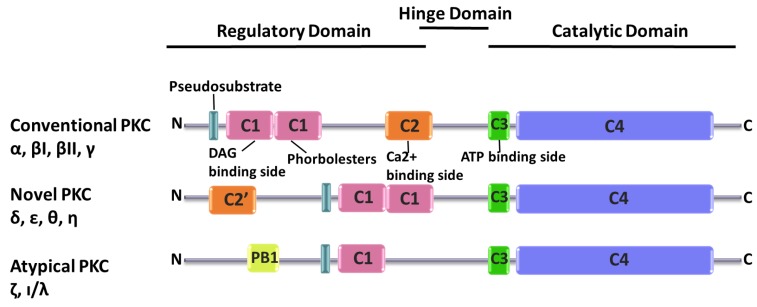
Domain structures of Protein kinase C (PKC) isozymes. C1 domain (pink), C2 domain (orange), C3 domain (green), Pseudosubstrate (turquoise), C4 domain (blue) and PB 1 (Phox and Bem1) domain (yellow).

Based on their structural features and activators, PKCs are classified into three categories: Conventional PKCs, atypical PKCs, and novel PKCs (Figure 1). Conventional PKCs consist of PKCα, PKCβI, PKCβII, and PKCγ. Conventional PKCs require DAG, phorbol esters (PE), and calcium for activation. Novel PKCs consist of PKCδ, PKCη, PKCθ, and PKCε. Compared to conventional PKCs, novel PKCs do not require Ca^2+^ for activation, but instead they need DAG and PE for the initiation of signaling cascades. Finally, atypical PKCs consist of PKCζ and PKC ι/λ, which only need DAG for activation.

Due to the regulatory role in cellular activities through interaction of other signaling proteins, PKCs are highly associated with disease progression.

## 3. Autophagy

The discovery of lysosomes by C. de Duve in the 1950s brought with it questions about the crucial cellular system including, what the fate across the regulation of lysosome is and how its regulation affects the protein-enzyme function [36]. During the investigation of these questions, another research team, Marilyn Farquhar and her associates focused on electron microscopy images of cells and discovered the existence of several membrane-covered vesicles. Initially, they described them as lytic bodies but subsequently, after these vesicles were observed engulfing mitochondria, ER and ribosomes they were defined as autophagic bodies [37]. Investigations have been accelerating and so far, autophagosomes, autolysosomes, and even different types of autophagic mechanisms have been discovered.

**Figure 2:**
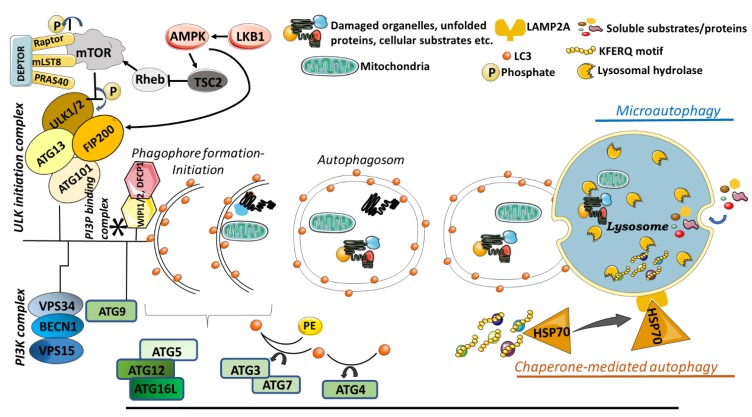
Illustration of main autophagy pathways. In macroautophagy, cellular contents are engulfed by double membrane vesicles called as autophagosome and carried to lysosome for degradation. In microutophagy, cytosolic components are directly taken into lysosome by lysosomal membrane invagination and degraded. In CMA, chaperon guiding proteins recognize target proteins and carry them to lysosome for degradation. mTOR: mammalian target of rapamycin protein, a highly conserved serine/threonine kinase, ULK1: Unc-51 Like Autophagy Activating Kinase 1, ULK2: Unc-51 Like Autophagy Activating Kinase 2 kinase, FIP200: focal adhesion kinase-family interacting protein of 200 kDa, PI3K: Complex of class III phosphatidylinositol 3-kinases, PI3P: Phosphatidylinositol-3-phosphate, AMPK: AMP-activated kinase, LKB1: liver kinase B1, Rheb: Ras-related small G protein, TSC2: tuberous sclerosis complex 2, VPS34: vacuolar protein sorting 34, VPS15: vacuolar protein sorting 15, BECN1: Beclin-1, PE: phosphatidylethanolamine, LC3: microtubule-associated protein light chain 3, WIPI: WD repeat domain phosphoinositide-interacting protein, DFCP1: Double FYVE-containing protein 1. In the figure, arrows indicate activation, whereas bars show inhibition.

Today, there are three main types of autophagy, namely macroautophagy, microautophagy and chaperone mediated autophagy (CMA) (Figure 2). In macroautophagy, larger molecules such as long-lived organelles or misfolded protein aggregates that could not be degraded by the proteasomal system are degraded and recycled. While; smaller molecules are directly transported to the lysosome for degradation via microautophagy, in chaperone-mediated autophagy, proteins carrying the KFERQ motif are first recognized by a chaperon protein, Heat Shock Protein 70 (HSP70), directed to lysosome and further recognized by lysosomal membrane protein 2A (LAMP2A) for engulfment and degradation by lysosomes [38].

### 3.1. Macroautophagy

Macroautophagy is the best characterized type of autophagy [39]. The mammalian target of rapamycin (mTOR) protein, a highly conserved serine/threonine kinase, negatively regulates the activation of autophagy. Indeed, there are two different mTOR complexes in mammalian cells: mTORC1 and mTORC2. mTORC1 is involved in cellular events such as cell growth, proliferation, and death [40,41,42], while mTORC2 regulates the cellular skeleton [43,44,45]. The mTORC1 complex integrates signals from nutrient sensing and growth factor pathways to regulate cell growth, protein synthesis and autophagy according to the availability of cellular resources.

The mTORC1 protein cluster, which plays a role in autophagy activation under starvation conditions, consists of proteins called Raptor, mLST8, PRAS40, and DEPTOR, which bind to mTor. When nutrient or growth factors are abundant, autophagy protein Ulk1 is phosphorylated by mTOR and this phosphorylation results in the inactivation of autophagy. The ULK complex contains the ULK1 or ULK2 kinase, ATG13, FIP200 (a focal adhesion kinase-family interacting protein of 200 kDa) and ATG101. However, when nutrients and growth factors are limited by the environment or under ER stress, AMP-Kinase (AMP-activated kinase) activated by LKB1 (liver kinase B1) via the phosphorylation of the Raptor directly on the mTORC1 cluster. Alternatively, AMP-Kinase inhibits mTORC1 by blocking of the Rheb (Ras-related small G protein) pathway via TSC2 inhibition in the upregulated signaling pathway of mTORC1. Thus, the formation of autophagosomes is triggered. When activated, mTORC1 favors cell growth by promoting translation via the phosphorylation of p70S6K (70 kDa polypeptide 1 ribosomal protein S6 kinase) and of 4E-BP1, an inhibitor of translation initiation, therein inactivating it [46].

Another protein complex needed for the formation of autophagosomes is a complex of class III phosphatidylinositol 3-kinases (PI3K). The core components of PI3K complex are also responsible for the catalytic activity of the complex and these are the VPS34 (vacuolar protein sorting 34), VPS15 and Beclin-1 (BECN1) proteins. PI3P plays a crucial role in the clustering of proteins required for autophagic vesicle formation in vesicle budding regions and provides a fusion platform for many proteins [47]. Phosphatidylinositol-3-phosphate (PI3P) constitutes an essential membrane component of the elongating isolation membrane and it is generated by VPS34 [48]. In mammalian cells, PI3P molecules function as a recruiting point for several autophagy-related proteins to the isolation membrane such as WIPI1/2 (WD repeat domain phosphoinositide-interacting protein), DFCP1 (double FYVE-containing protein 1), and Alfy [49]. Additionally, ATG9 is also one of the crucial transmembrane proteins, as it plays a role in lipid delivery and establishes a platform of recruiting effectors for the phagophore [50].

The autophagic sac elongation involves two separate protein-protein interactions, which resemble ubiquitination. The first pathway enables the binding of the ubiquitin-like ATG12 protein to ATG5 and then the ATG5-ATG12 complex recruits ATG16L to form the ATG12-ATG5-ATG16 complex. Here, the ATG7 and ATG10 proteins, which regulate the addition of ATG12 in a manner similar to ubiquitination, play the E1 and E2-like role, respectively [51]. The second pathway is the covalent binding of phosphatidylethanolamine (PE) to the protein LC3. LC3B protein is synthesized as a precursor and is a critical component of autophagosome formation. During this formation, cysteine protease ATG4 cleaves LC3 from its C-terminus and a glycine residue is exposed. The cleaved form of LC3 is activated by ATG7, then transferred to ATG3 through covalent binding and linked to an amino group of phosphatidylethanolamines (PE). The conjugation of LC3-PE to both sides of the isolation membrane enables it to act as a surface receptor for the specific recruitment of other proteins [52]. The autophagosomal closure results in a typical double-membraned vacuole formation.

Apart from AMP-Kinase, PTEN, one of the tumor suppressors, converts PIP3 to PIP2 and inhibits mTOR via PI3K/Akt/TSC1-2 pathway so that has a role in autophagy regulation. In addition, ERK1/2 and c-Jun N-terminal kinase1 (JNK1) in Ras/MAPK pathway have been found to be inducers of autophagy [53,54]. Some oncogenes such as Class I PI3K, Akt, TOR, Bcl-2 suppress autophagy. Studies have shown that p53, one of the most important tumor suppressor genes, plays a dual role in autophagy [55,56]. A number of studies have shown that p53, particularly in the cell nucleus, induces autophagy, either dependently or independently of the effect of transcription [57,58]. On the other hand, some studies suggest that wild and mutant forms of p53 located in the cytoplasm suppress autophagy [59,60]. In addition, TNF-α (Tumor necrosis factor), which plays a fundamental role in many disease mechanisms, including cancer, has been shown to activate mTORC1 by phosphorylating the TSC1 complex (IKKβ) [61].

### 3.2. Microautophagy

Microautophagy involves direct engulfment of the proteins/cytoplasm into the lysosome (mammalians) or vacuole (plants and fungi) by invagination. Dynamin-related GTPase Vps1p plays an active role in regulating the invagination of microautophagic process. Importantly, these invaginations grow and shrink rapidly, and their frequency depends on nutritional conditions. Starvation induces the initiation and extension of invagination respectively [62]. During the extension, this formation specializes into a characteristic tubular shape termed the “autophagic tube”, depending on its unique structure and autophagy-related function [63].

The soluble substrates of microautophagy can be induced by N-starvation or rapamycin through the regulatory signaling complexes. The maintenance of organelle size, membrane homeostasis, and cell survival under N-restriction are considered the main functions of microautophagy [64].

As a basic form of autophagy, the microautophagy-dependent lysosomal/vacuolar degradative process is either non-selective or selective. The non-selective microautophagy engulfs soluble intracellular substrates by tubular invagination, while the selective microautophagy sequesters specific organelles with arm-like protrusions. The non-selective microautophagy is regularly observed in mammalian cells, while the three forms of selective microautophagy are frequently induced in yeasts (micropexophagy, piecemeal microautophagy of the nucleus (PMN), micromitophagy).

### 3.3. Chaperone Mediated Autophagy

Chaperone mediated autophagy (CMA) is a selective form of autophagy and it has a distinctive way to recognize cargo molecules. In CMA, the well-known chaperone protein heat shock protein 70 (hsp70) recognizes proteins with specific pentapeptide motif KFERQ and translocates them to the lysosomal lumen for degradation via lysosomal-associated membrane protein 2A (LAMP2A) [65]. CMA activation is crucial for some cellular events such as control of the cell cycle [66], CD4+ T cell activation [67], protein quality control [68], and as a response to starvation [69]. In addition, a link between CMA mechanism and neurodegenerative diseases has been reported. It is known that, CMA contributes to the degradation of misfolded proteins, which are prone to become aggregates.

### 3.4. Selective Autophagy

**Figure 3:**
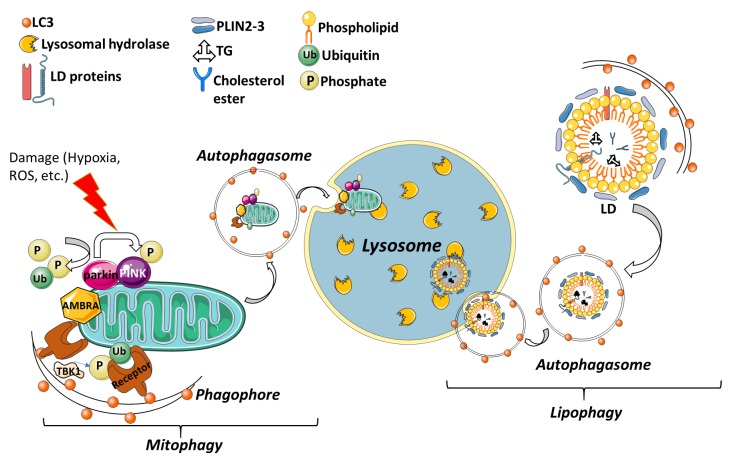
Selective autophagy degrades nonfunctional organelles and lipid droplets engulfed by double membrane vesicles. Non-functional or damaged mitochondria or lipid droplets are enclosed by phagophore then they are directed through lysosome for recycling.

For cellular homeostasis, the proper clearance of organelles or specific molecules is critical in living organisms. In selective autophagy, receptor proteins recognize specific cargo such as mitochondria, lipid droplets, invading pathogens etc. (Figure 3). These receptor proteins are responsible for carrying the cargo to the site of autophagosomal engulfment. So far, several specific receptors or adaptor proteins, that regulate the selective degradation of specific cargo have been identified and partially characterized. Selective autophagy has drawn the attention of researchers because of its potential importance in clinical diseases; however, its physiological roles are not yet fully understood.

#### 3.4.1. Mitophagy

Mitochondria are highly dynamic, double membrane surrounded organelles, which play a major role in energy production within eukaryotic cells. They are involved in a variety of cellular functions within eukaryotic cells and have an ancient bacterial origin. Besides energy production, they are involved in amino acid synthesis, fatty acid production, heme synthesis, innate immunity [70], as well as programmed cell death processes [71].

Considering all these functions of the mitochondria, the ability to control its balance according to cellular demand is essential for disease progression. The selective degradation to remove damaged mitochondria by autophagy is called mitophagy [72]. Parkin and PINK1-dependent mitophagy is one of the best-studied forms of mitophagy [73,74]. Mainly, mitophagy is maintained by these two famous genes and their loss-of-function mutations are linked to early-onset Parkinson’s Disease. PTEN-induced putative kinase 1 (PINK1), encodes for a mitochondrially localized kinase, and PARK2, encodes a cytosolic E3 ubiquitin ligase [74]. Under normal conditions, after being synthesized as a precursor in the cytoplasm, PINK1 is imported to the mitochondria through translocase of the outer membrane (TOM) and translocase of the inner membrane (TIM) complexes. When PINK1 is imported, it is post-translationally modified within the mitochondria by mitochondrial proteases. PINK1 is first cleaved through its N-terminal matrix targeting sequence (MTS) by matrix processing peptidases (MPP) and resulting in cleavage followed by another cleavage by Presenilin-associated rhomboid-like protease (PARL) in the matrix [75]. PARL-mediated N-terminal cleavage results in the destabilizing of Phe104 residue and therefore, when retranslocated from mitochondria to cytoplasm it emerges and degrades by proteasome through recognition of its N-terminus [76]. Under stress conditions, PINK1 import to mitochondria is blocked and PINK1 proteins on OMM are dimerized and this dimerization is necessary for autophosphorylation events. This accumulated PINK1 phosphorylates various targets, including ubiquitin, and recruits the cytoplasmic E3 ubiquitin ligase, Parkin protein. From then on, Parkin acts as an amplifier of the PINK1-generated mitophagy signal [77].

In addition, AMBRA1 is another key mitophagy regulator that allows proper Parkin-dependent and independent mitochondrial clearance. It is ubiquitously expressed in the adult midbrain and is found in complex with Parkin but AMBRA1 has no effect on Parkin recruitment to mitochondria, and it has been suggested that its activator effect on PI3K is critical for PINK/Parkin-mediated mitophagy [78].

#### 3.4.2. Lipophagy

Lipid droplets (LDs) are specialized organelles composed of lipids essential for cellular energy (metabolism) and membrane production [79]. LDs consist of a neutral lipid namely triglyceride (TG) and cholesterol esters and are coated by a phospholipid monolayer and various proteins such as perilipins (PLINs). Under certain conditions, such as nutrient deprivation, cellular lipids stored as triglycerides in LDs are hydrolyzed into fatty acids for energy. As expected, the second cellular response to starvation is the induction of autophagy. Interrelationship between autophagy and lipid metabolism was shown in mouse hepatocytes for first time in 2009 by Mochida et al. This study revealed that the inhibition of macroautophagy, pharmacologically or by silencing of the ATG genes, leads to the accumulation of TGs and LDs in serum-starved hepatocytes. Thus, nutrient limitation triggers LD degradation by macroautophagy in hepatocytes [80].

Microlipophagy has been detected in yeast in response to nitrogen starvation, glucose depletion, survival during the stationary phase, and phospholipid imbalance. Under these conditions, LDs are taken up into the vacuole at sites of vacuolar membrane invagination [81,82].

## 4. PKC in Autophagy Mechanism

**Figure 4:**
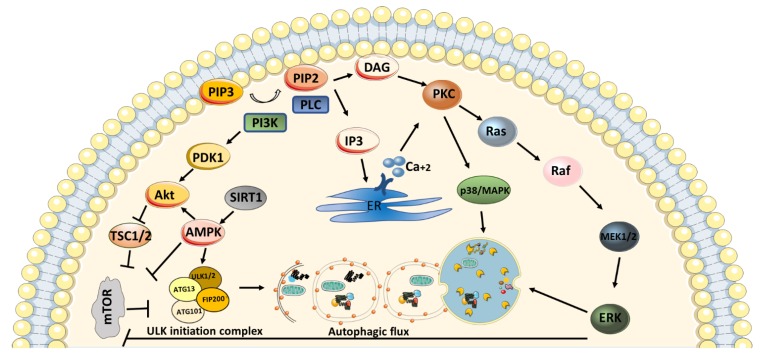
Schematic representation of main signaling pathways playing role in autophagy activation. In the figure, arrows indicate activation, whereas bars show inhibition. Activation of phosphatidylinositol 3-kinase (PI3K) results in dephosphorylation of phosphatidylinositol [3,4,5]-trisphosphate (PIP3) and generates phosphatidylinositol (4,5)-bisphosphate (PIP2). Phospholipase C (PLC) hydrolyses PIP2 and produces inositol-1, 4,5-trisphosphate (IP3) and diacylglycerol (DAG). IP3 translocates to endoplasmic reticulum (ER) in order to release calcium. DAG and calcium binding activate protein kinase C (PKC). Activation of PKC stimulates downstream signaling pathways; mitogen-activated protein kinase (p38/MAPK) and RAS/RAF/MEK/ERK pathways for autophagy regulation. Also, PI3K mediates initiation of Akt/mammalian target of rapamycin (mTOR) signaling pathway for inhibition of autophagy by suppressing Unc-51 Like autophagy activating kinase (ULK) complex. Activation of adenosine monophosphate-activated protein kinase (AMPK) and silence information regulator 1 (SIRT1) causes activation of autophagy through both suppression of mTOR or activation of Akt or ULK initiation complex.

The activation of Protein kinase C isozymes is involved in extensive cellular mechanisms, including the autophagy pathway. Each isozyme has a diverse role in the pathway due to its phosphorylation abilities. Various functions of PKCs in main autophagy pathways such as PI3K/Akt /mTOR/Erk have been reported in several studies [83,84,85,86] (Figure 4).

It has been suggested that, PKCα suppresses autophagy by inducing expression of miR-129-2 in maternal diabetes and provokes neural tube defects [87]. In another study, tetrandrine, a PKCα inhibitor, was shown to cause autophagy induction in breast cancer cells through the AMPK independent and mTOR dependent mechanism [88]. On the other hand, Xue et al. showed that the inhibition of PKCα causes lysosomal dysfunction and abnormalities in autophagosome-lysosome fusion in NRK-49F cells. Thus, the recovery of autophagic flux by PKCα activation resulted in kidney fibrosis through TGFβ-1 induction [89]. Cisplatin, a chemotherapeutic agent, plays a role in initiating several signaling pathways in order to activate cell death. The expression of PKCβ is promoted by cisplatin treatment in HeLa cells. When PKCβ is silenced, suppression of cisplatin-induced apoptosis was observed while the formation of cisplatin-induced autophagy was promoted [90]. Another drug, clozapine used in the treatment of schizophrenia, has a role in autophagy regulation. PKCβ restriction by ruboxistaurin increases clozapine-induced lipophagy and causes the recycling of lipid accumulation both in vitro and in vivo [91]. Conventional PKCγ reduces neuron-specific autophagy via phosphorylation of mTOR on serine 2448 and serine 2481 [92]. It has been reported that cPKCγ provokes the inhibition of ubiquitin C-terminal hydrolase L1 (UCHL1) and is involved in the ERK-mTOR mediated autophagy during ischemic neural injury [93].

Moreover, the inhibition of PKCδ plays a role in the correction of nephrotoxicity in kidneys by upregulating autophagy through blockage of cisplatin-induced mTOR, Akt, and ULK1 pathway [94]. Similarly, PKCδ was used to phosphorylate GSK3αβ and suppress autophagy under cadmium-induced heme oxygenase-1 (HO-1) expression [95]. Inhibition of PI3K/Akt/mTOR pathway through PKCδ results in the impairment of autophagic flux in neural retina cells [96]. PKCε has a role in lipid metabolism by participating in hepatocyte autophagy [97]. In reverse, PKCε suppresses autophagy in glioblastoma cells [98]. Another isozyme, PKCθ, activation in Epstein-Barr virus (EBV)-infected cells causes phosphorylation of p38/MAPK, where it leads to autophagy induction [99]. Hypoxic-induced autophagy is enhanced under calcium-dependent PKCθ activation [100]. Interestingly, under pathogenic infection, autophagy mechanism is upregulated due to an increase in the pathogen’s phagosomal escape, and PKCη silencing results in the suppression of ATG7. Even though ATG7 levels decreased in the absence of PKCη, the number of phagosomes is not changed. Therefore, PKCη in autophagy regulation may have indirect effects and is suggested for further research [101].

PKCι downregulates autophagic flux by repressing LC3 conversion. Accordingly, PKCι knockdown resulted in autophagic degradation of Hsc70 through CMA independent mechanism [102]. Also, downregulation of PKCι promotes autophagic degradation of β-catenin, independent of CMA [103]. In another study, PKCι knockdown induces autophagy via the restriction of PI3K/Akt/mTOR pathway. Overexpression of mutant PKCι protein induces autophagosome formation and depletion in p62 protein. Therefore, it has been suggested that overexpression of mutant PKCs may be used as antagonists of wild type PKCι for the upregulation of autophagy [104].

## 5. Autophagy in Neurodegenerative Diseases

### 5.1. Parkinson’s Disease

Parkinson’s disease (PD) is one of the age-related neurodegenerative disorders that occurs due to the accumulation of Lewy bodies in dopaminergic neurons. To date, several genes have been identified as a bridge between Parkinson’s disease and autophagy as a protective mechanism for the clearance of toxic protein aggregation. In Drosophila models, Parkinson’s specific genes, Fbxo7 and Parkin, involve in the CCCP induced mitophagy. In the absence of Parkin, Fbxo7 does not change the degradation of mitochondria, while in the presence of Parkin, the degradation occurs. Therefore, Fbxo7 regulates mitophagy through Parkin dependent manner [105]. Leucine-rich repeat kinase 2 (LRRK2), an autosomal dominant gene of PD, has an impact on lysosomal degradation through p62. p62 has a binding site at the N-terminal region of LRRK2, and overexpression of p62 affects the autophagic degradation of LRRK2 [106]. Comparably similar studies showed that LRRK2 interacts with different targets as leucyl-tRNA synthetase [107], nicotinic acid adenine dinucleotide phosphate [108], dynamin-like protein 1 [109] and activation of MEK/ERK pathway by MAPK/ERK kinases phosphorylation [110]. Wild-type α-synuclein protein in Parkinson’s disease is efficiently degraded by CMA. Yet, pathogenic alpha synucleins accumulate and inhibit CMA activation [111]. DJ-1, a protein encoded by PARK7 gene, regulates autophagy mechanisms in several ways; where its inhibition causes the activation of the JNK pathway, and the increase in p62 degradation [112]. DJ-1 mutation causes a defect in mitochondria through changing its morphology [113,114] and affects Parkin dependent autophagy in PD. It has been indicated that, expressed DJ-1 plays a role in α-synuclein degradation via CMA since α-synuclein aggregation is induced in knockdown DJ-1, both in vitro and in vivo. Likewise, DJ-1 deficiency causes a decrease in autophagic flux and degradation of α-synuclein in microglia [115]. The decline in the CMA regulation occurs with the increase in the LAMP2A degradation in lysosomes under the inhibition of DJ-1. The results are specific solely for LAMP2A as inhibition of DJ-1 does not change the LAMP1 level [116]. In addition, early stage PD patients have decreased protein level of LAMP2, which indicates that, CMA dysfunction is an early step of PD development [117]. 6-hydroxydopamine (6-OHDA) is a synthetic neurotoxin utilized to generate PD models and its treatment causes the secretion of DJ-1 due to oxidative stress and phosphorylation of AMPK and activation of AMPK/ULK1 pathway-dependent autophagy [118].

On the other hand, Ivatt et al. studied on a genome-wide RNAi screen in order to discover possible target genes on autophagy mechanism during Parkinson’s disease progression. They found 20 genes that play a role in Parkin mediated mitophagy and significantly, the suppression of SREBF1, FBXW7 and GSK3A genes cause direct inhibition of Parkin translocation to mitochondria. In the end, the absence of translocation resulted in the inhibition of mitophagy [119].

### 5.2. Alzheimer’s Disease

Alzheimer’s disease, the most common specific form of dementia, is a progressive neurodegenerative disease characterized by gradual memory fragmentation, impaired cognitive abilities, disruptions in daily living activities, and psychological and behavioral changes [120].

There are two groups in Alzheimer’s disease, Early-Onset Alzheimer’s Disease (familial form) and Late-Onset Alzheimer’s Disease (sporadic form). Genetic mutations with autosomal dominant inheritance (APP, PSEN1, and PSEN2) are present in the familial form, while genetic variations and multifactorial risks (education, quality of life, income, nutrition, etc.) are common in the sporadic form which accounts for more than 90% of Alzheimer’s disease cases [121,122,123]. As in Parkinson’s disease, mitochondrial dysfunction and autophagy have a vital role in disease development. The molecular pathology of AD is characterized by as neural cell death, the accumulation of amyloid-β (Aβ) and tau proteins in neurons and microglia. Similar to PD, the mutant tau protein that generates amyloidogenic products, are not engulfed by lysosomes. Incomplete translocation of mutant tau leads to its partial cleavage into highly amyloidogenic peptides, which form irreversible oligomers at the lysosomal membrane [124]. Mitophagy causes the disease to retreat through the increase in the turnover of protein aggregation and by the adequation of cellular energy metabolism. In the subjects, some of the mitophagy proteins as phospho-ULK1, phospho-TBK1 and BNIP3L/NIX are lessened so that the defective mitochondria could not be degraded properly [125]. Accumulation of Aβ causes an impediment in the axonal transport and results in autophagic stress in axons. The interaction of Aβ with DIC impacts dynein-snapin complex formation and elevates retrograde transport to increase the autophagy mechanism in axons [126]. Accumulation of Aβ alters the mTOR signaling and causes a defect in learning and memory. APP mutant mice as AD model shows high mTOR activity compared to control however APP/mTOR +/− mutant mice show the reduction in mTOR activity but not complete blockage. Due to be a negative regulator of autophagy, reduction in mTOR initiates the induction of the autophagy mechanism. In APP/mTOR +/− mutant mice, the expression of autophagic proteins is higher than APP mutant mice so the accumulation of amyloid and other AD-related proteins can be degraded by mTOR mediated autophagic pathway [127]. SIRT1 activation also affects the tau phosphorylation and Aβ-induced GSK3β activation, therefore, it can be used as a block in neurodegeneration and improvement of learning and memory [128]. Amyloid β and APP aggregation can be disposed of by cilostazol-induced autophagy in neurons. Aβ accumulation causes the decrease in some of the autophagic proteins as BECN1 and ATG5 however cilostazol treatment reverses the Aβ effect on autophagy activity through SIRT1 protein [129]. Mutant BECN1 mice show unremitting activation in autophagy mechanism either under stress or basal conditions. Their AD progression is suppressed by the inhibition of Beclin and Bcl2 interaction [130].

### 5.3. Amyotrophic Lateral Sclerosis (ALS)

Amyotrophic lateral sclerosis (ALS) is characterized as a neuromuscular disorder that arises from defects in the motor neurons. The most causative gene for ALS development is Cu/Zn superoxide dismutase (SOD1) and mutations in this gene cause the accumulation of impaired mitochondria in axons as well as damage to the autophagy recycling mechanism. fALS-linked mutant SOD mice shows lysosomal deficiency in early asymptomatic stage since mutant SOD acts as an antagonist of dynein during retrograde transport and hinders dynein-snapin complex formation. In fALS-linked mutant SOD mice, p62 accumulation follows the colocalization of p62 with hSOD1G93A; however, the protein could not be degraded due to lysosomal deficiency. The dynein-snapin complex has a pivotal role in the autophagy mechanism in axons, therefore the blockage of the complex results in the accumulation of abnormal organelles and proteins [131]. ATG7 cKO; SOD1G93A double mutant mice show a reduction in p62 accumulation and interestingly prolong the lifespan of mice [132]. P2X7 overexpression encourages autophagic protein in SOD1G93A primary microglia and causes formation of ALS pathogenesis [133]. On the other hand, rilmenidine, disseminates the autophagy mechanism through the mTOR independent pathway and induces the degradation of SOD1G93A and p62 aggregates. In SOD1 mutant mice, there is an increase in LAMP2A level and it colocalizes with SOD1. However, after rilmenidine treatment, LAMP2A colocalization with SOD1 did not occur. Therefore, rilmenidine may affect autophagy flux via CMA independent manner [134].

### 5.4. Huntington’s Disease

In Huntington disease (HD), an extension of polyQ (polyglutamine) tract in the N-terminus of Huntingtin (HTT) protein causes protein aggregation. Accordingly, the accumulation of mutant HTT protein results in the induction of autophagic flux. In most neurodegenerative diseases, autophagic activity is suppressed; however, in this aspect, HD follows a different in particular by inducing selective macroautophagy. Starting within the research of how polyQ tract is related with autophagy, it has been found that loss of polyQ tract even in wild type HTT proteins causes longer neural activity in mouse models, suggesting the potential of clinical actionability upon autophagic pathway upregulation [135]. Through the research on structural similarities and homology studies of HTT protein, selective autophagy protein Atg11 emerged as a potential partner with common binding interactions, referring that HTT may typically function as a scaffold for several types of selective autophagy. Furthermore, central nervous system studies in mouse and Drosophila models showed protein accumulation in the absence of HTT function [136,137].

In selective autophagy, HTT protein is in charge by physically interacting the autophagy cargo receptor p62, whereas acts as an inducer by binding to the kinase that initiates autophagy, ULK1 [138]. In HD, cargo trafficking in autophagic flux is impaired based upon a failure of autophagosome motility regulation, therefore preventing fusion with the lysosome [139]. Besides the projected roles of HTT protein, HTT protein is also in charge in the regulation of motility during cargo trafficking, starting from mitochondria to various vesicles [140].

### 5.5. Multiple Sclerosis (MS)

Multiple sclerosis (MS) is a clinically diverse autoimmune disease of the central nervous system, resulting in continual neurological impairments as a subset of the chronic inflammatory demyelinating disorders. With respect to of autophagy, MS progression strictly links since autophagy mechanism acts as the maintainer for the structure of mitochondria. Accordingly, the dysfunction prevention of mitochondria is a critical checkpoint in order to avoid cell death and MS progression. Suppression and loss of autophagy, which results in higher protein accumulation, is associated with neurodegenerative diseases in mice models [141]. In the central nervous system, complex and mutual regulation between inflammatory response and autophagy is present where each element plays as interactors for each other. For instance, ATG9 acts as an inflammatory response reducer by modulating over type-I IFN signaling [142].

Another example might show that, Atg16L1 lowers inflammatory immune responses in mice model [143]. Yet, the autophagic activity becomes a regulator for adaptive immune responses. For instance, during the development of T-cell MHC-II–peptide repertoires, autophagy favors tolerance and a wide array of antigens, regardless of certain tissue specificity [144].

Besides the immune responses, autophagy is linked to MS in the processes of demyelination and remyelination. For instance, under rapamycin influence, a potent autophagy inducer, autophagy reduces PMP22 aggregates, therefore improving myelination in TrJ mice [145,146].

## 6. Roles of PKC Isozymes in Neurodegenerative Disease Progression via Autophagy Mechanism

Presently, we have demonstrated the PKC-dependent autophagy in the course of neurodegenerative diseases. PKCα has a crucial role in neural tube defects (NTD) in embryonic mouse models. It induces miR-129-2 and inhibits peroxisome proliferator-activated receptor c coactivator 1 (PGC-1) expression. The inhibition of PGC-1 impairs autophagy and causes NTD formation. In diabetic pregnancies, the overexpression of PKCα causes impairment in the autophagy mechanism through PGC-1 and results in NTD [87]. Also, PGC-1 overexpression protects neurons from mitochondrial dysfunction under oxidative stress in Parkinson’s disease [147].

Another study shows that inhibition of PKCα induces autophagic activity through activation of TGFβ-1, therefore promoting the differentiation and growth of axons by the phosphorylation of Par6 [148]. PKCs have ability to enhance phosphorylation of Par6 and TGFβ and then induce cell migration of neurons [149]. Par6 phosphorylation is an important event in cell polarization and embryonic inactivation. Accordingly, polarization causes severe conditions such as, the imbalance in neural development. The Par3-Par6-aPKC complex has an evolutionary conserved mechanism to maintain embryonic development in eukaryotic organisms. Also, it plays a fundamental role in axon expansion and multiple axon formation [150]. mTOR controls cell survival under growth-promoting factors and it has the ability to phosphorylate cPKC and Akt in order to establish protein stability [151]. The inhibition of ubiquitin C-terminal hydrolase L1 (UCHL1) by cPKCγ restricts autophagy through the activation of ERK-mTOR pathway and reduces ischemic neural injury [93].

Tetramethylpyrazine (TMP) induces the phosphorylation of Erk, Akt and PKCζ and promotes the migration of neural precursor cells (NPC). Inhibition of PKC by Myr-ψPKC causes the blockade of TMP mediated NPC migration [139]. Nagai studied milk phospholipids’ (mPL) effects on the endoplasmic reticulum (ER) stress-mediated autophagy in Neuro2A cells, and according to theirresults, the activation of PKC isozymes by mPL induces the protective effect of neurons under ER stress conditions with the increase in the autophagy mechanism [152]. The protective impact of PKCγ on hypoxia/ischemia [153] and the PKCγ mediated neural autophagy through Akt/mTOR pathway under ischemic stroke were studied [154]. Mitochondrial neurotoxin MMP+ promotes autophagic cell death and both JNK and Akt/mTOR signaling pathways are activated. When apoptosis specific JNK protein increased, cellular survival proteins, Akt and mTOR, levels diminished under dose dependent MMP+ treatment. However, when the cells treated with PKC agonist TPA, the destructive effect of MMP+ eliminated [155].

Autophagic flux tightly intertwines with innate and adaptive immune systems. Since autophagy is a crucial player for the fate of a cell in mitochondrial dysfunction, it acts upon in MS progression [156]. Increasing points of evidence in MS pathogenesis comprise that cortical demyelination is a consequence of the production of reactive oxygen species (ROS). In this aspect, PKC has a role in populating ROS by particularly activating NADPH oxidase. Resulting nitrative and oxidative stress will potentially lead to cell death [157]. Furthermore, PKC-β serine phosphorylates p66, which will eventually contribute to targeting isomerase Pin1 to oxidize cytochrome c in the mitochondrial intermembrane space [158]. Accordingly, reduced oxygen into ROS will cause cell death through permeability transition pore (PTP) opening [159].

It has been shown that PKC-λ/ι enhances the production of brain β-secretase, Aβ1, and phospho-tau and cause hyperinsulinemia in Alzheimer’s disease. Importantly, the inhibition of brain αPKC restores the increase in the β-secretase and Aβ production and also repairs the impairment in memory function due to high fat diet in mice [30,160].

Most neurodegenerative disorders are linked with misfolded protein aggregation and impaired degradation mechanisms are highly present. Besides autophagy, there are several other degradation mechanisms in the cell, and they have specific reporters in order to label target proteins for recycling. One of reporters is named as small ubiquitin-like modifier (SUMO) and its activation induces SUMOylation [161]. PKCα induces SUMOylation of metabotropic glutamate receptor subtype 7 (mGluR7) via phosphorylation at Ser862 and improves its stability on cell membrane therefore causes a decrease in the receptor endocytosis. Phosphorylation of mGluR7 by PKC provokes synaptic plasticity in neurons [162,163].

Activation of mGluR7 improves the cell viability, proliferation and differentiation of neural stem cells (NSCs). mGluR7 activation via phosphorylation regulates the survival of NSCs and the (PI3K)/Akt signaling pathway takes part in the activation. PKC is an intermediate agent between mGluR7 and (PI3K)/Akt and therefore it may have a critical role in the proliferation and differentiation of neural stem cells [164].

## 7. Polyphenols Act as Neuroprotective Agent During PKC and Autophagy Regulation

Phenolic and flavonoid compounds are secondary metabolites of plants and have been heavily used in alternative and complementary medicine for decades [165]. Extraction and analysis of these compounds have shown that at the optimum doses they could act as neuroprotective agents, while overdosages cause damage in neurons [166,167]. There are several studies which indicate that different polyphenols and small compounds alter the PKC regulation by phosphorylation and result in disease protection. Here, we noted the small compounds’ role in both PKC and autophagy signaling on neurodegenerative disease pathogenesis (Table 1). Also, we screened current therapeutic agents used in neurodegenerative diseases linked with PKC signaling (Table 2).

Epigallocatechin 3-gallate (EGCG), which is one of the phenolic compounds of green tea, has been reported as a protective agent [168,169,170,171]. EGCG influences autophagy to maintain cell survival under ER stress [172] and decrease the prion accumulation mediated neurodegenerative disease formation by stimulating autophagic flux through SIRT1 activation [173]. EGCG also acts as a neuroprotective agent by inhibiting mitochondrial dysfunction and recovery of impaired autophagy under subarachnoid hemorrhage stroke [174]. EGCG treatment in neuroblastoma cells causes phosphorylation of PKC and activation of the nonamyloidogenic α-secretase pathway of APP against Aβ neurotoxicity [175] and EGCG dependent PKC activation induced neuroprotection under 6-OHDA exposure in cells [176]. Moreover, it has been reported that EGCG protects neuron cells under stress-induced brain impairment by promoting PKCα and ERK1/2 signal pathways [177].

The grape polyphenolic compound resveratrol is another neuroprotective agent against Aβ induced neuronal toxicity [178,179]. Resveratrol plays a role in the reduction of Aβ toxicity by inducing autophagy mechanism via AMPK/SIRT1 pathway in neuroblastoma cells [180] and acts as a protective factor against cerebral ischemia through stimulation of AMPK dependent autophagy pathway [181]. It has been reported that resveratrol has direct interaction with the mTOR complex and the inhibition of mTOR results in the initiation of autophagy [182]. It promotes PKC phosphorylation and reduces Aβ toxicity in hippocampal cells [183]. Resveratrol binding to the C1 domain of PKCα results in PKC inactivation. As PKCα, Munc13-1, one of the presynaptic proteins regulating neurotransmitter release, has a resveratrol binding region on C1 domain [184]. Both bindings of resveratrol on PKCα and Munc13-1 inhibits neurotoxicity.

Quercetin treatment plays a neuroprotective role in brain injuries by inducing neuronal autophagy and inhibiting cell death through PI3K/Akt signaling pathway [185]. Quercetin also has a role in rotenone-induced neurotoxicity in PD. Moreover, quercetin treatment reverses impaired autophagic activity and results in the protection of the brain against oxidative stress [186]. Furthermore, quercetin reduces PKCε/p38MAPK mediated ROS production through the activation of the ERK1/2 pathway to protect neurons from oxidative stress [187] and it is indicated that GSK-β and p38 inhibition or PKC activation results in Nrf2 phosphorylation under quercetin treatment [188]. Suppression of PKCε and TRPV1 in the spinal cord by quercetin has the potential for the treatment of acute neuropathic pain [189]. Also, PAR2/TRPV1 signaling inhibition by quercetin causes a decrease in PKCγ expression as well as depletion in inflammatory markers in bone cancer models [190].

Another natural compound, curcumin, decreases neuronal cell death under oxidative stress by inducing autophagy and inhibiting ROS production [191]. On the contrary, curcumin has neuroprotective effects on cerebral ischemia-reperfusion through reducing autophagy via PI3K/Akt/mTOR pathway [192]. It has been reported that the inhibition of calcium channels by curcumin treatment is connected with PKCθ signaling and promotes neuroprotection in brain ischemia against an increase in intracellular calcium concentration [193].

## 8. Conclusions

In this review, we discussed how autophagic pathways and their interaction with PKCs play a role in the development of neurodegenerative diseases. Today, it is known that different types of autophagic pathways potentially play a great role in the maintenance and ultimately the fate of cell. Similarly, some of the PKC isozymes are known as intermediate modulators or agents for the autophagic pathway/neuronal fate while some remain mysterious.

The role of PKCs in autophagy signaling during neurodegenerative disease development may constitute a triangle. Considering the complex workings of the brain and its astounding ability to adapt and overcome disability, the interaction of these pathways may have promising therapeutic potential for the treatment of neurodegenerative diseases in the future.

## Figures and Tables

**Table 1:** Natural compounds’ effects on protein kinase C isozymes and autophagy.

PKC Isozymes	Compound	Function
PKCα PKCε	Epigallocatechin 3-gallate (EGCG) [169,170,171,172,173,174,175,176,177]	Neuroprotection against Aβ toxicity Promoting autophagy
PKCδ PKCα	Resveratrol [182,183,184]	Attenuating cellular toxicity Inducing autophagy mechanism via AMPK/SIRT1 and mTOR
PKCε PKCδ PKCγ	Quercetin [185,186,187,188,189]	Inducing neuronal autophagy and inhibiting cell death through PI3K/Akt signaling Decreases neuropathic pain
PKCθ	Curcumin [191,192,193]	Increase autophagy in neuroblastoma Decrease autophagy under cerebral ischemia- reperfusion via PI3K/Akt/mTOR

**Table 2:** Small molecule modulators and their targets involved in neurological diseases with respect to Protein kinase C (PKC) isozymes.

Disease	Molecule	Class/SubClass	Target	Mode of Action	Targeted Pathway	Affected PKCs Isozyme	PKC Related Action	Clinical Trial Status
Parkinson’s	Levodopa [194]	Carboxylic acids—Amino acids, peptides	Dopamine D1, D2, D3, D4, D5 receptors	Agonist	Dopaminergic synapse	PKC-α, PKC-β, PKC-γ	Induced phosphatidylinositol signaling	FDA Approved 2008
	Rasagiline [195]	Benzenoids—Indanes	Amine oxidase (flavin-containing) B, Apoptosis regulator Bcl-2	Inhibitor	Serotonergic synapse	PKC-α, PKC-β, PKC-γ	Neuroprotection by inhibiting Caspase 3	FDA Approved 2006
	Bromocriptine [196]	Ergoline—Lysergic acids	D2 dopamine receptor	Agonist	Neuroactive ligand-receptor interaction	PKC-α, PKC-β, PKC-γ, PKC-ε	Enhanced MAPK phosphorylation and PKC activity	FDA Approved 2004
	Pramipexole [197]	Organonitrogen—Amines	Dopamine D2, D3, D4 receptors	Agonist	Dopaminergic synapse	PKC-α, PKC-β, PKC-γ	PKC/MAPK pathways interference	FDA Approved 1997
	Quetiapine [198]	Benzothiazepines—Dibenzothiazepines	D2 dopamine receptor, 5-hydroxytryptamine receptor 2A	Antagonist	Neuroactive ligand-receptor interaction	PKC-α, PKC-β, PKC-γ	PKC activation through cAMP and calcium pathways	Phase IV Completed
	Isradipine [199]	Benzoxadiazoles	Calcium voltage-gated channel subunit alpha1 C	Inhibitor	GABAergic synapse	PKC-α, PKC-β, PKC-γ	Increased PKC activity	Phase III Completed
	Pimavanserin [200]	Phenol ethers	D2 dopamine receptor, 5-hydroxytryptamine receptor 2A	Inverse Agonist	Neuroactive ligand-receptor interaction	PKC-α, PKC-β, PKC-γ	PKC activation and phosphatidylinositol signaling	Phase III Completed
Alzheimer’s	Memantine [201]	Organonitrogen—Amines	Glutamate ionotropic receptor NMDA type subunit 3A	Antagonist	Neuroactive ligand-receptor interaction	PKC-α, PKC-β, PKC-γ	Reduced depolarization-induced phosphorylation of PKC	FDA Approved 2003
	Galantamine [202]	Piperidines—Benzylpiperidines	Acetylcholinesterase	Inhibitor	Cholinergic synapse	PKC-α, PKC-β, PKC-γ	Calmodulin-dependent PKC activation	FDA Approved 2001
	Rivastigmine [203]	Benzene—Phenoxy compounds	Acetylcholinesterase	Inhibitor	Cholinergic synapse	PKC-α, PKC-β, PKC-γ	Stimulation of sAPPα increases PKC activity	FDA Approved 2000
	Rosiglitazone [204]	Phenol ethers	Peroxisome proliferator-activated receptor gamma	Agonist	PPAR signaling pathway	PKC-α, PKC-β, PKC-γ	Downstream modulation of PKC through adenylyl cyclase	FDA Approved 1999
	Donepezil [205]	Piperidines—Benzylpiperidines	Acetylcholinesterase	Modulator	Cholinergic synapse	PKC-α, PKC-β, PKC-γ	Activation of phospholipase C / PKC	FDA Approved 1996
	Aripiprazole [206]	Diazinanes—Piperazines	D2 dopamine receptor, 5-hydroxytryptamine receptor 2A	Antagonist	Dopaminergic synapse	PKC-α, PKC-β, PKC-γ	Reduced phosphorylation of DRD2	Phase IV Completed
	TRx0237 [207]	Benzothiazines	Microtubule-associated protein tau	Aggregation Inhibitor	MAPK signaling pathway	PKC-α, PKC-β, PKC-γ	Decreased activity of PKC through tau inhibition	Phase III Completed
	Nilvadipine [208]	Pyridines—Hydropyridines	Voltage-dependent L-type calcium channel subunit alpha-1C	Inhibitor	GABAergic synapse	PKC-α, PKC-β, PKC-γ	Reduced activation via PKC	Phase III Completed
	Intepirdine [209]	Diazinanes—Piperazines	5-hydroxytryptamine receptor 4	Antagonist	Neuroactive ligand-receptor interaction	PKC-δ	Decreased activity of PKCδ through c-src kinase	Phase III Completed
	Idalopirdine [210]	Indoles—Tryptamines	5-hydroxytryptamine receptor 2A	Antagonist	Neuroactive ligand-receptor interaction	PKC-α, PKC-β, PKC-γ	Reduced calmodulin mediated phosphorylation	Phase III Completed
	Brexpiprazole [211]	Diazinanes—Piperazines	5-hydroxytryptamine receptor 1A, D2 dopamine receptor	Agonist/Partial agonist	Neuroactive ligand-receptor interaction	PKC-α, PKC-β, PKC-γ	Increased phosphorylation activity	Phase III Completed
	Atorvastatin [212]	Pyrroles—Substituted pyrroles	3-hydroxy-3-methylglutaryl-coenzyme A reductase	Inhibitor	AMPK signaling pathway	PKC-α, PKC-β, PKC-γ	Reduced phosphorylation of HMGCR	Phase III Completed
Multiple Sclerosis	Fingolimod [213]	Organonitrogen—Amimes	Sphingosine 1-phosphate receptor 5	Modulator	Neuroactive ligand-receptor interaction	PKC-βII	Increased PKC activity	FDA Approved 2010
	Cannabidiol [214]	Prenol lipids—Monoterpenoids	Cannabinoid receptors	Agonist	Neuroactive ligand-receptor interaction	PKC-α, PKC-β, PKC-γ	Hippocampal PKC/neurogranin signaling	Phase IV Completed
	Teriflunomide [215]	Benzene—Trifluoromethylbenzenes	Dihydroorotate dehydrogenase	Inhibitor	Metabolic pathways	PKC-δ	PI3-kinase/PKC-δ and nuclear factor-kappa B signaling	Phase IV Completed
	Ponesimod [216]	Phenol ethers	Sphingosine-1-phosphate receptor 1	Modulator	Neuroactive ligand-receptor interaction	PKC-α, PKC-β, PKC-γ	Inhibited PKC signaling	Phase III Completed
	Ozanimod [217]	Azoles—Oxadiazoles	Sphingosine-1-phosphate receptor 5	Modulator	Neuroactive ligand-receptor interaction	PKC-ε	Increased PKC-ε activity	Phase III Completed
	Duloxetine [218]	Naphthalenes	Sodium-dependent dopamine transporter	Inhibitor	Dopaminergic synapse	PKC-ε	PKC-ε activity through cytokines release	Phase III Completed
	Arbaclofen [219]	Carboxylic acids—Amino acids, peptides	GABA type A receptor associated protein like 1	Inhibitor	GABAergic synapse	PKC-α, PKC-β, PKC-γ	Reduced PKC phosphorylation	Phase III Completed
Huntington’s	Deutetrabenazine [220]	Tetrahydroisoquinolines	Solute carrier family 18 member A2	Inhibitor	Dopaminergic synapse	PKC-α, PKC-β, PKC-γ	Reduced PKC phosphorylation	FDA Approved 2017
	Tetrabenazine [221]	Tetrahydroisoquinolines	D2 dopamine receptor	Inhibitor	Dopaminergic synapse	PKC-β	Reduced dopaminergic signaling	FDA Approved 2015
	Riluzole [222]	Benzothiazoles	Glutamate metabotropic receptor 1	Inhibitor	Neuroactive ligand-receptor interaction	PKC-α, PKC-β, PKC-γ	Inhibited PKC signaling	Phase III Completed
	Tiapride [221]	Benzene—Benzenesulfonyl	D2 dopamine receptor	Inhibitor	Dopaminergic synapse	PKC-β	Reduced dopaminergic signaling	Phase III Completed
	Pridopidine [223]	Piperidines—Phenylpiperidines	Superoxide Dismutase-1	Modulator	Neuroactive ligand-receptor interaction	PKC-α, PKC-β, PKC-γ	Stimulation of PKC activity	Phase III Completed
	Minocycline [224]	Tetracyclines	Caspase-3	Inhibitor	Serotonergic synapse	PKC-α, PKC-βII	Downregulated MHC II through PKC Inhibition	Phase III Completed
	Olanzapine [225]	Benzodiazepines	D2 dopamine receptor	Agonist	Dopaminergic synapse	PKC-α, PKC-β, PKC-γ	Enhanced PKC activity	Phase III Completed
ALS	Edaravone [226]	Azolines—Pyrazolines	Peroxyl radicals	Inhibitor	ROS-Triggered Intracellular Signaling	PKC-α, PKC-β, PKC-γ	Enhanced PKC activity	FDA Approved 2017
	Riluzole [222]	Benzothiazoles	Glutamate metabotropic receptor 1	Inhibitor	Neuroactive ligand-receptor interaction	PKC-α, PKC-β, PKC-γ	Inhibited PKC signaling	FDA Approved 1995
	Mexiletine [227]	Phenol ethers	Sodium channel protein type 5 subunit alpha	Inhibitor	Adrenergic signaling in cardiomyocytes	PKC-α, PKC-βI	Enhanced PKC activity	Phase IV Completed
